# Supplementary material for: Carbohydrate-binding protein from stinging nettle as fusion inhibitor for SARS-CoV-2 variants of concern
Source: Front Cell Infect Microbiol. 2022 Aug 30;12:989534. doi: 10.3389/fcimb.2022.989534 (PMC9468479; doi:10.3389/fcimb.2022.989534)
Supplement: Supplementary file 1 [file DataSheet_1.pdf]

Supplementary Material

Supplementary Material

1 Supplementary Figures

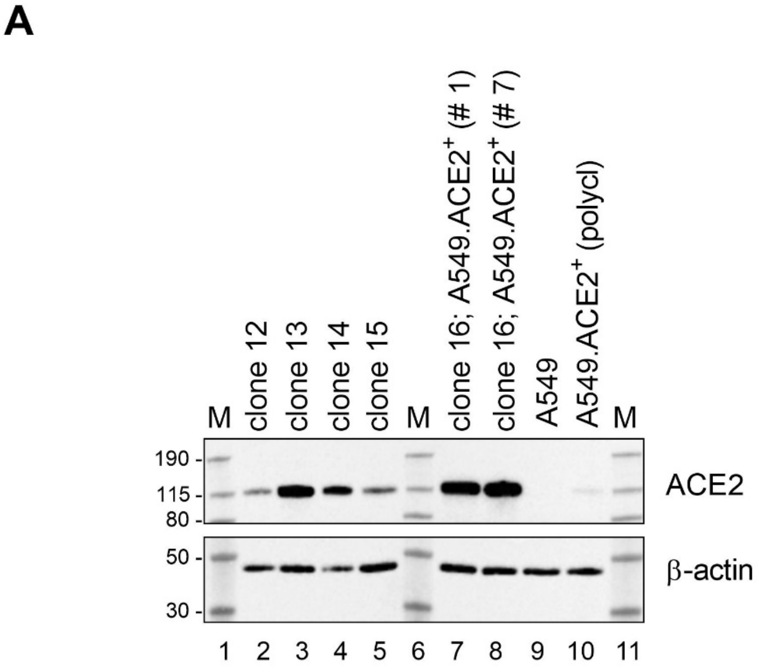

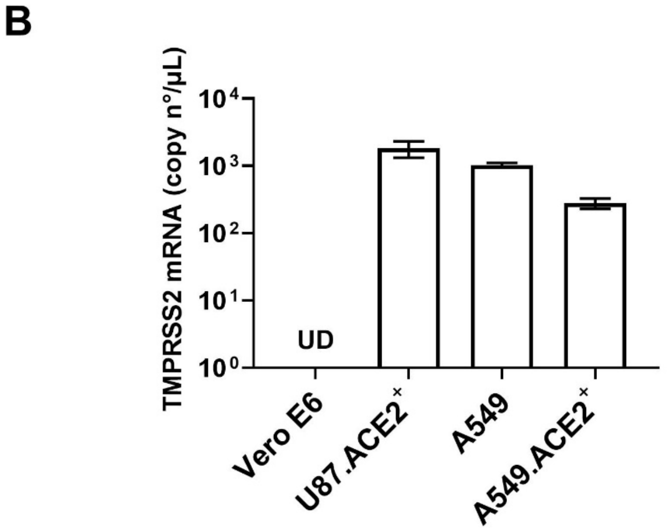

Supplementary Figure 1. Stable expression of ACE2 in transduced A549.ACE2+ cells. (**A**) Different clones of A549 cells were analysed for ACE2 expression by immunoblotting, with β-actin as loading control. Clone 16 was selected for this study. The stably ACE2 transduced A549 cells (clone 16) were

tested at an early (# 1) and later (# 7) passage of the cells (lanes 7 and 8, respectively). The parental A549 cells did not express detectable levels of ACE2 (lane 9). The polyclonal mixture (polycl) of ACE2-transduced A549 cells is also included (lane 10), showing some expression of ACE2. M; molecular marker in kDa. (**B**) Comparative qPCR analysis of TMPRSS2 mRNA levels between the stably ACE2 transduced A549 cells (clone 16) and Vero E6, U87.ACE2^{+} and the parental A549 cells. Graph shows copy numbers/µl as calculated from a TMPRSS2 standard. The TMPRSS2 level in Vero E6 cells was below detection limit. Data are collected from 2 biological replicates analysed in duplo (mean ± SD; n =4).

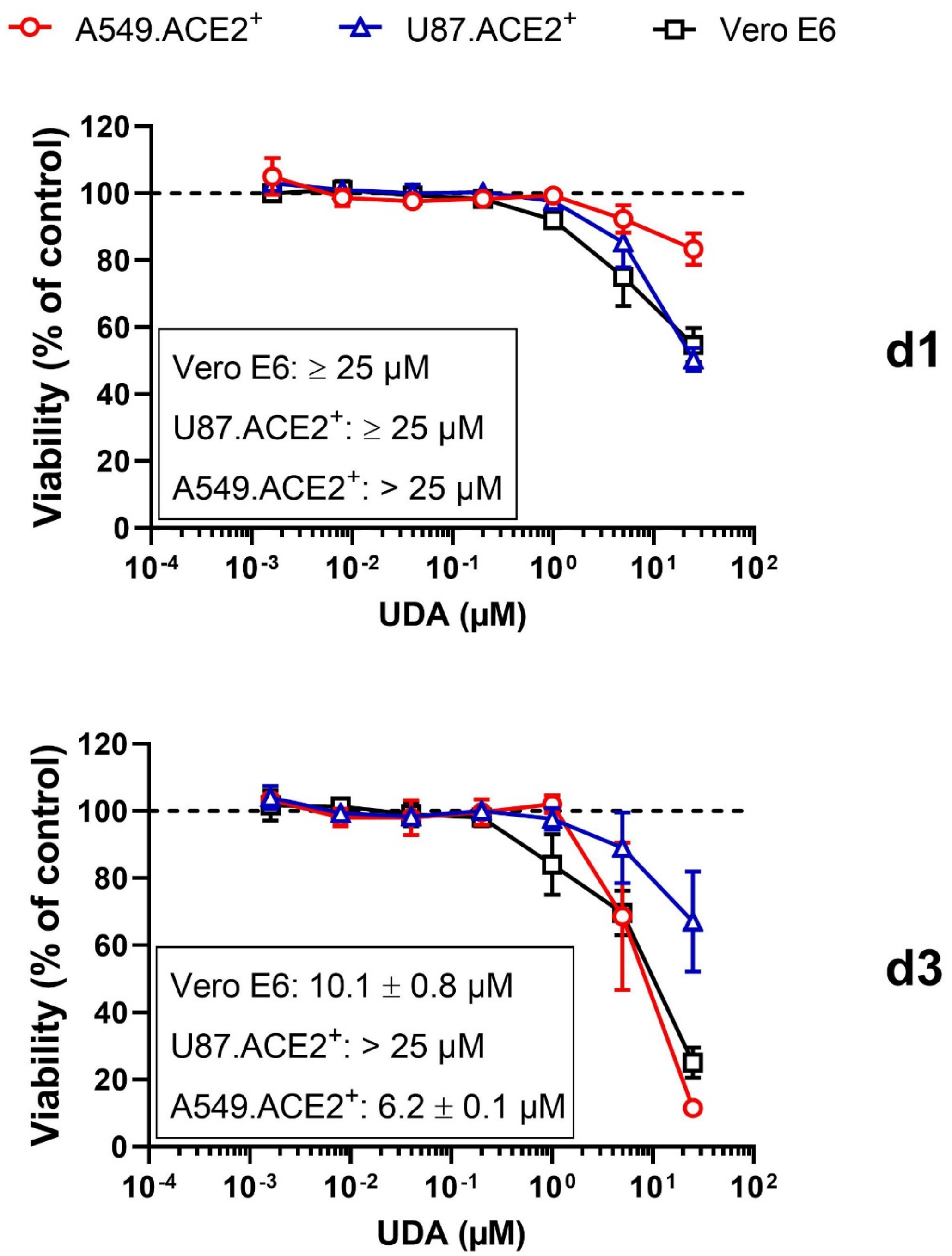

Supplementary Figure 2. Cytotoxicity of UDA. Cells were seeded in 96-well microtiter plates and exposed to 5-fold serial dilutions of UDA (starting from 25 µM). After an incubation of one (top panel) or three (bottom panel) days, the cell viability of the cells was assessed spectrophotometrically with MTS. Graph represents a concentration-response of UDA from 3 independent experiments (mean ± SD; n=3). For each experiment, the concentration that induced cell death by 50% (CC_{50}) was calculated from interpolation and the average value of 3 experiments is given in the insert (mean ± SD; n=3).

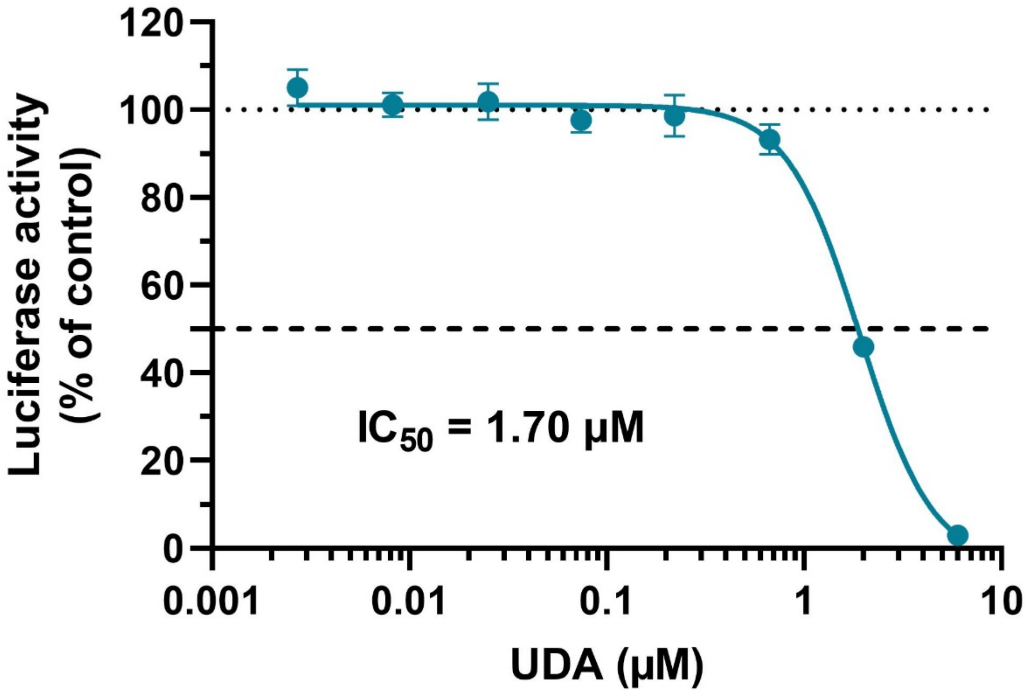

Supplementary Figure 3. Antiviral activity of UDA against pseudotyped Delta SARS-CoV-2. UDA was tested against luciferase-based pseudotyped SARS-CoV-2 (expressing the spike from the Delta VOC) in commercially available A549.ACE2^{+}.TMPRSS2^{+} cells. At 22h after VLP transduction, luciferase activity was measured. Graph represents a concentration-response of UDA from 2 biological replicates in quadruple (mean ± SD; n=8).

A

Ligand/Analyte	k_a (x 10^5 $M^{-1}s^{-1}$)	k_d (x 10^{-5} s^{-1})	K_D (nM)
Wuhan-Hu-1 spike/UDA (n=8)	3.37 ± 0.22	240.43 ± 13.03	7.37 ± 0.92
Omicron spike/UDA (n=4)	3.49 ± 0.20	364.55 ± 9.25	10.58 ± 0.82
Wuhan-Hu-1 RBD/UDA (n=2)	3.10 ± 0.60	709.50 ± 221.50	22.35 ± 2.85
Wuhan-Hu-1 RBD/R001 (n=3)	19.70 ± 0.87	4.17 ± 0.17	0.020 ± 0.001
Wuhan-Hu-1 RBD/R007 (n=6)	2.93 ± 0.37	16.80 ± 0.89	0.66 ± 0.15
Wuhan-Hu-1 RBD/R007 (n=2)*	4.00 ± 0.77	15.25 ± 1.35	0.39 ± 0.04

B

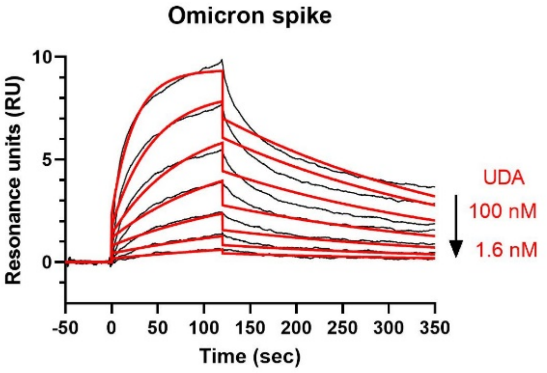

C

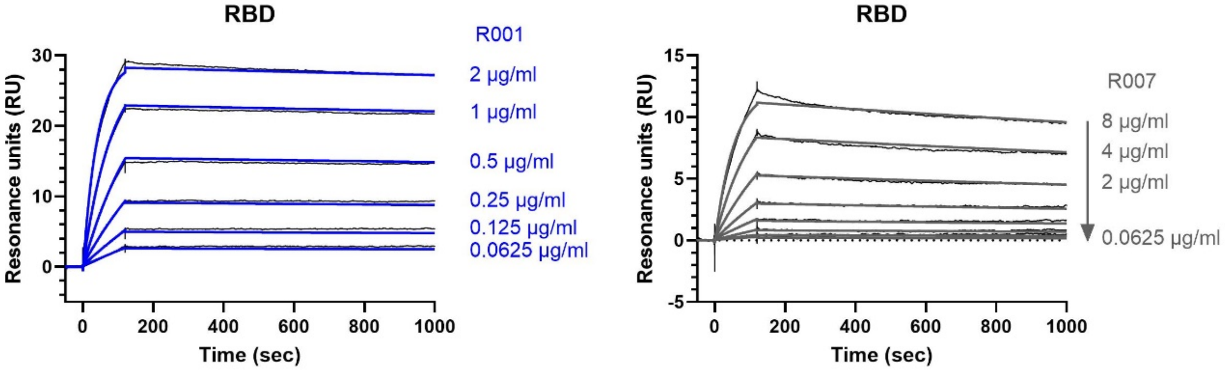

Supplementary Figure 4. Surface plasmon resonance (SPR) analysis of UDA and spike-binding antibodies R001 and R007. (**A**) Summary of the kinetics parameters from different repeat SPR experiments performed in this study. Given are the association rate constant (k_a), the dissociation rate constant (k_d), and the dissociation equilibrium constant (K_D). Values are mean ± SEM. (*) For the

interaction of R007 to RBD, two different sensor chips were used, an NTA chip (n=8) or an CM5 chip (n=2). (**B**) SPR sensorgram showing the binding kinetics for UDA and immobilized monomeric Omicron spike protein (1:2 dilutions of UDA, starting from 100 nM). Data are shown as black lines, and the best fit of the data to a 1:1 binding model is shown in red. (**C**) SPR sensorgrams showing the binding kinetics for spike-binding antibodies and immobilized RBD of Wuhan-Hu-1 spike. Left panel shows sensorgram for the spike-neutralising antibody R001 and right panel that of the non-neutralising spike-binding antibody R007. Data are shown as black lines, and the best fit of the data to a 1:1 binding model is shown in blue or grey, respectively. SPR: surface plasmon resonance; RBD: receptor binding domain; UDA: *Urtica dioica* agglutinin; NTA: nitrilotriacetic acid.

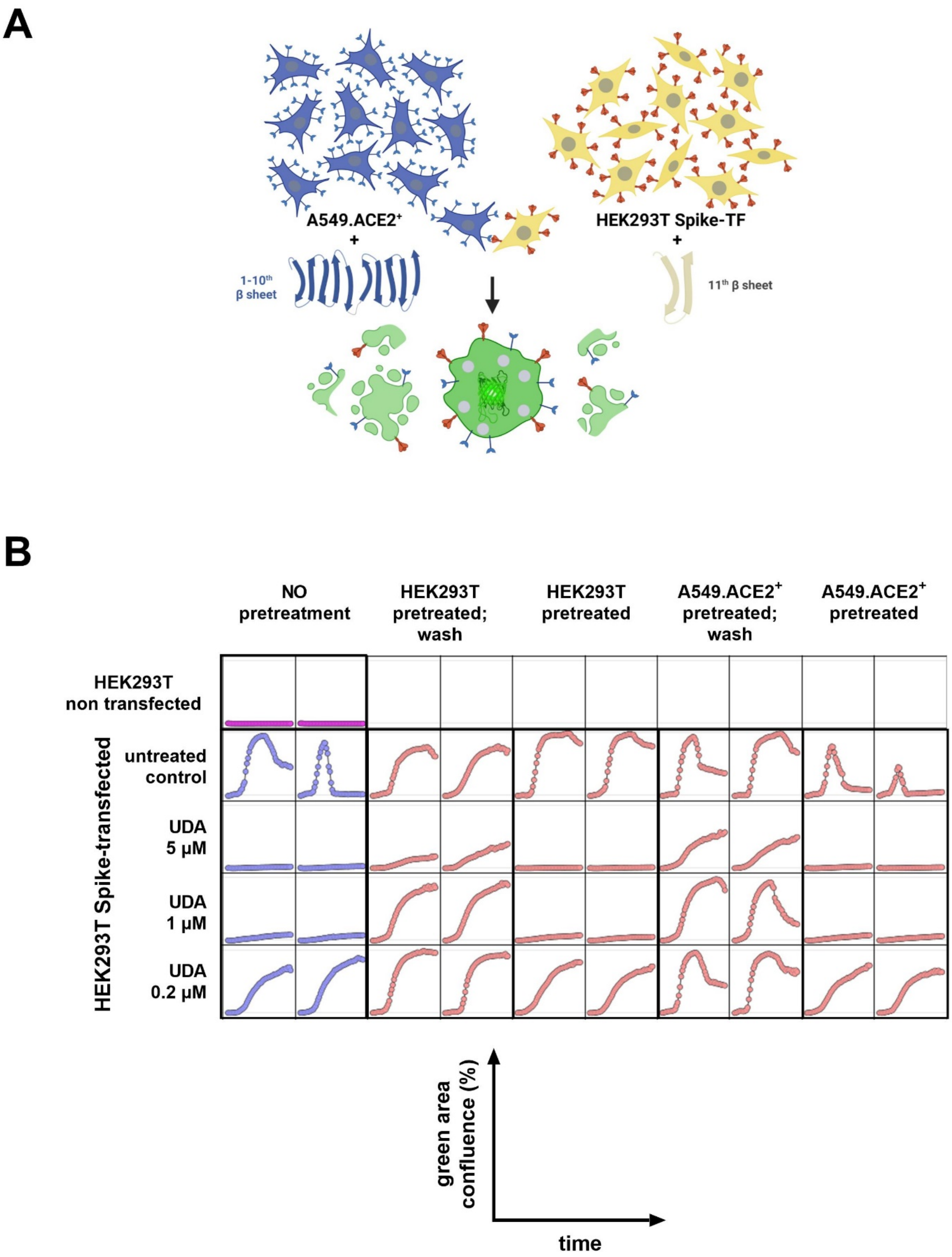

Supplementary Figure 5. UDA prevents cell-cell fusion of A549.ACE2^{+} cells with spike-expressing HEK293T cells. (**A**) A549.ACE2^{+} cells (transfected to express the first 10 betasheets of neongreen) were overlayed with HEK293T cells co-transfected with a plasmid encoding the SARS-CoV-2 spike protein and a plasmid encoding the 11th betasheet of neongreen. Only cell-cell fusion of an A549 cell

with a HEK293T cell will result in the assembly of a functional neongreen protein and give a green fluorescence signal. (**B**) Samples from Figure 5 were analysed for neongreen expression (for specification of the samples, see legend to Figure 5). Each condition was tested in 2 replicate wells (side-by-side columns), and in each well 4 different areas of the cell culture were monitored using an Incucyte live-cell analysis instrument. The increase in neongreen expression over time (17 hours) is plotted as percentage of green area confluence. Each individual plot shows the average signal of the 4 different areas of the cell culture (mean; n=4). Note that because of lysis of the syncytia, the fluorescent protein is diluted in the culture medium, resulting in a drop in the neongreen signal at later time points.

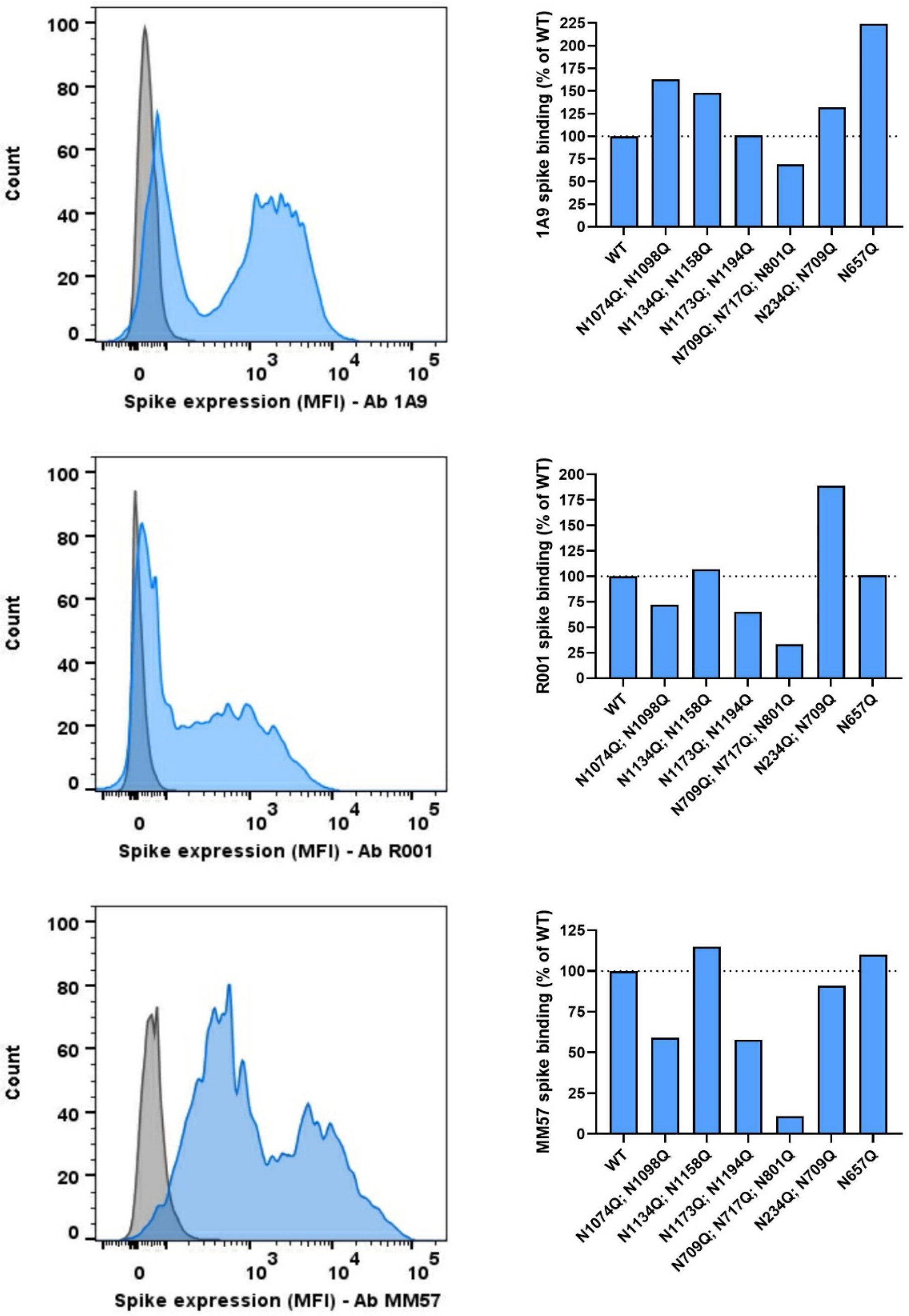

Supplementary Figure 6. Cell surface spike expression of different N-glycosylation mutants. HEK293T cells were transfected with a plasmid encoding the wild-type (WT) SARS-CoV-2 spike protein or an N-glycosylation deletion mutant. At 24h post transfection, cells were collected and stained with three different anti-spike antibodies (as indicated) to determine the cell surface expression of S.

Histogram plots on the left show the mean fluorescence intensity (MFI) of S protein expression for non-transfected (grey) and WT S-transfected (blue) HEK293T cells. Flow cytometric data were collected from approximately 6,000 analyzed cells. The bar graphs on the right show S expression for the different spike mutants as determined by staining with the corresponding anti-spike antibody (as indicated). Bars represent the MFI relative to the WT control.

2 Supplementary Movie

Supplementary movie. A549.ACE2$^+$ cells (transfected to express the first 10 betasheets of neongreen) were overlayed with HEK293T cells co-transfected with a plasmid encoding the SARS-CoV-2 spike protein and a plasmid encoding the 11th betasheet of neongreen. Overlay was done in the absence (untreated control; left) or presence of UDA (1 µM; right). Fusion events were visualized using the IncuCyte® S3 Live-Cell Analysis System (Sartorius). Phase contrast and GFP images were taken using a 20x objective lens at 20 minute intervals for a 24 hours period. Image processing was performed using the IncuCyte software.
